# Physiologically Relevant In Vitro-In Vivo Correlation (IVIVC) Approach for Sildenafil with Site-Dependent Dissolution

**DOI:** 10.3390/pharmaceutics11060251

**Published:** 2019-06-01

**Authors:** Tae Hwan Kim, Soyoung Shin, Seok Won Jeong, Jong Bong Lee, Beom Soo Shin

**Affiliations:** 1 School of Pharmacy, Sungkyunkwan University, Suwon, Gyeonggi 16419, Korea; thkim@cu.ac.kr (T.H.K.); jswpia1@naver.com (S.W.J.); 2 College of Pharmacy, Wonkwang University, Iksan, Jeonbuk 54538, Korea; shins@wku.ac.kr; 3 Department of Pharmaceutics, Ernest Mario School of Pharmacy, Rutgers, The State University of New Jersey, Piscataway, NJ 08854, USA; myjblee@gmail.com

**Keywords:** sildenafil, in vitro-in vivo correlation, site-dependent dissolution, pharmacokinetics, population pharmacokinetic modeling

## Abstract

This study aimed to establish a physiologically relevant in vitro-in vivo correlation (IVIVC) model reflecting site-dependent dissolution kinetics for sildenafil based on population-pharmacokinetic (POP-PK) modeling. An immediate release (IR, 20 mg) and three sustained release (SR, 60 mg) sildenafil tablets were prepared by wet granulation method. In vitro dissolutions were determined by the paddle method at pH 1.2, 4.5, and 6.8 media. The in vivo pharmacokinetics were assessed after oral administration of the prepared IR and SR formulations to Beagle dogs (*n* = 12). The dissolution of sildenafil from SR formulations was incomplete at pH 6.8, which was not observed at pH 1.2 and pH 4.5. The relative bioavailability was reduced with the decrease of the dissolution rate. Moreover, secondary peaks were observed in the plasma concentration-time curves, which may result from site-dependent dissolution. Thus, a POP-PK model was developed to reflect the site-dependent dissolution by separately describing the dissolution and absorption processes, which allowed for estimation of the in vivo dissolution of sildenafil. Finally, an IVIVC was established and validated by correlating the in vitro and in vivo dissolution rates. The present approach may be applied to establish IVIVC for various drugs with complex dissolution kinetics for the development of new formulations.

## 1. Introduction

Sustained release (SR) oral dosage formulations are designed to make a drug available for absorption over an extended period after administration. Thus, SR formulations could lead to reduced dosing frequency, minimized unwanted side effects, and improved patient compliance, which overall would enhance the therapeutic effects. Nevertheless, the development process of SR formulations is usually costly and time-consuming. The major difficulty in the successful development of SR formulations is that it often requires multiple non-clinical and clinical studies to demonstrate the desired pharmacokinetics of the SR formulation compared to its reference immediate release (IR) formulation. It is particularly difficult if the drug possesses complex pharmacokinetic characteristics due to the unusual absorption behaviors, such as pH-dependent or site-specific dissolution or absorption. For example, since drugs may have variable solubilities depending on pH, the dissolution rates of the drug may be varying in different regions of the gastrointestinal tract. Consequently, such pH-dependent dissolution characteristics can result in unusual pharmacokinetic profiles, such as the second or shoulder peaks in the plasma concentration versus time profiles or reduced bioavailability with the decrease in release rates. This can lead to difficulties in demonstrating equivalence in maximum concentration (C_max_) and area under the plasma concentration-time curve (AUC) between the SR and the reference formulations, providing one of the main hurdles for the development of new SR formulations.

According to the FDA guidance, in vitro and in vivo correlation (IVIVC) is defined as a predictive mathematical model describing the relationship between an in vitro property of a dosage form and an in vivo response [1]. Thus, IVIVC can be used to predict the in vivo pharmacokinetics of the SR formulation from its in vitro dissolution data. In contrast, it can also help to design the optimal in vitro dissolution profiles of the SR formulation for the desired in vivo pharmacokinetics. Therefore, once the predictability of IVIVC has been established, the in vitro dissolution may provide a surrogate for in vivo experiments. Among the different levels of IVIVC, a level-A IVIVC, which allows for predicting the entire in vivo plasma concentration-time course from the in vitro data, is most useful to justify a biowaiver [1,2].

During the establishing of IVIVC, extracting the in vivo input profile, which directly corresponds to the in vitro dissolution profile, is one of the critical steps. Although Wagner-Nelson [3], Loo-Riegelman [4], and numerical deconvolution methods have been utilized to deconvolute the in vivo plasma concentration versus time profiles to estimate the in vivo input profiles for highly permeable drugs, the use of these conventional approaches may be limited for drugs with complex dissolution or absorption processes [5,6,7]. This is because the in vivo dissolution and absorption cannot be separated and only the combined consecutive dissolution and absorption can be estimated by the conventional methods. Especially, if a drug that exhibits complex dissolution or absorption kinetics and each process is significantly affected by physiological factors along the gastrointestinal tract, the estimation of the in vivo input profile from the plasma pharmacokinetics is even more challenging. 

Recently, various modeling techniques, such as population pharmacokinetics (POP-PK) [5,6,8] and physiologically-based pharmacokinetics (PBPK) [9,10] modeling, have been applied to develop IVIVC for drugs that have poor predictability with the conventional IVIVC methods. The advantages of these modeling-based approaches are that they can separately describe the dissolution and absorption process and reflect various factors affecting each process. For example, our previous study has demonstrated the utility of POP-PK-based approaches to establish IVIVC for a biopharmaceutics classification system (BCS) class III drug, loxoprofen, with a site-specific absorption process [6]. 

The present study, therefore, aimed to develop an IVIVC approach that can be applicable for drugs with site-dependent dissolution characteristics by using POP-PK modeling. Sildenafil, a selective inhibitor of cyclic guanosine monophosphate (cGMP)-specific phosphodiesterase type 5, was used as a model drug. Sildenafil is known to be a BCS class II drug which has high permeability and low solubility, and is practically insoluble at a pH higher than 6 [11]. Since the solubility of sildenafil considerably varies within the physiological pH ranges of the gastrointestinal tract [11], the dissolution and absorption of sildenafil may not be consistent throughout the gastrointestinal tract, but significantly vary depending on the regional environment of the gastrointestinal tract. Thus, the dissolution and absorption kinetics of sildenafil SR tablets, which have different release rates, cannot be easily estimated through the conventional IVIVC approaches. In this study, we formulated one IR and three SR tablets of sildenafil and the in vivo pharmacokinetics were evaluated after oral administration in Beagle dogs. Based on the obtained plasma concentration versus time profiles of IR and SR tablets, in vivo dissolution of sildenafil in the gastrointestinal tract was extracted by POP-PK modeling. Finally, the estimated in vitro and in vivo dissolution rate parameters were correlated, establishing IVIVC. We believe that the present approach may be applied to establish IVIVC for various drugs which have site-dependent dissolution.

## 2. Materials and Methods 

### 2.1. Materials

Sildenafil citrate was obtained from KyungDong pharm. co., Ltd. (Seoul, Korea). Anhydrous lactose and sodium carboxymethylcellulose (Na CMC) were purchased from Whawon Pharm. Co. (Seoul, Korea). Hydroxypropyl methylcellulose (HPMC) 2208-100 cps and polyvinylpyrrolidone K30 were the products of Shin-Etsu Chemical Co., Ltd. (Tokyo, Japan). Tadalafil, an internal standard (IS) for the liquid chromatography-tandem mass spectrometry (LC-MS/MS) assay, was obtained from Sigma-Aldrich Co. (St. Louis, MO, USA). High-performance liquid chromatography (HPLC) grade ethanol, hydrochloric acid, and potassium dihydrogen phosphate were purchased from Merck Co. (Darmstadt, Germany). Acetonitrile and water (HPLC grade) were purchased from J.T. Baker Co. (Philipsburg, NJ, USA).

### 2.2. Formulation

Wet granulation method was used to prepare an IR (20 mg) and three different types of SR (60 mg) sildenafil tablets. The recommended dose of sildenafil for the treatment of pulmonary arterial hypertension is 20 mg three times a day. Compositions of the prepared formulations are summarized in Table 1. Three types of SR formulations were designed to present fast (SR_fast_), medium (SR_medium_), and slow (SR_slow_) drug release profiles. Lactose was used as a diluent and magnesium stearate was used as the lubricant for both IR and SR tablets. For the preparation of IR tablets, sodium carboxymethylcellulose and polyvinylpyrrolidone K30 were used as a disintegrant and a binder, respectively. To prepare SR tablets with different drug release rates, HPMC 2208-100 cps was used as a drug release modifier. Sildenafil citrate was mixed with the diluent and disintegrant and the mixture was kneaded with binder dissolved in 60% ethanol. The dampened mixture was then kneaded and passed through a size-20 mesh screen and dried at 60 °C for 60 min. The granules were then passed through a 1.4 mm sized mesh and magnesium stearate was added to the dried granules. The resulting lubricated granules were weighed and compressed at 2-ton force by a hydraulic tablet press (Carver, Inc., Wabash, IN, USA). 

### 2.3. In Vitro Dissolution Testing 

The in vitro dissolution test was performed by the paddle method using Distek Dissolution System 2500 coupled with the Evolution Dissolution Sampler 4300 (North Brunswick, NJ, USA). The dissolution media were 0.1 N HCl (pH 1.2), acetate buffer (pH 4.5), and phosphate buffer (pH 6.8). Medium temperature was maintained at 37 ± 0.5 °C, and the paddle stirring speed was fixed at 100 rpm. The samples (2 mL) were collected by an auto-sampler at 0.25, 0.5, 0.75, 1, 1.5, 2, 3, 4, 6, 8, 10, 12, 16, and 24 h. After collecting the sample, the sampled medium volume was refilled by fresh medium. All collected samples were filtered through a 45 μm polyethylene syringe filter (Distek, North Brunswick, NJ, USA). 

### 2.4. In Vivo Pharmacokinetic Study

The animal study was approved by the ethics committee for the treatment of laboratory animals at KNOTUS Co., Ltd., Guri, Korea on 4 September 2017 (KNOTUS IACUC 17-KE-243). Beagle dogs (12 male dogs, 14–18 months) were purchased from Orient Bio (Seongnam, Korea). After overnight fasting, the reference IR tablets containing 20 mg of sildenafil or the three different SR tablets containing 60 mg of sildenafil were orally administered with 30 mL of water to the Beagle dogs (*n* = 3, each). Blood samples (3 mL) were collected via the cephalic vein into a heparinized (5 IU/mL) tube at 0, 0.25, 0.5, 0.75, 1, 1.5, 2, 2.5, 3, 3.5, 4, 5, 6, 7, 8, 12, 24, 36, and 48 h following administration. Plasma samples were harvested by centrifugation of the collected blood samples at 4000 × g at 4 °C for 10 min and stored at –20 °C until analysis.

### 2.5. Analytical Methods

#### 2.5.1. HPLC

Sildenafil concentrations in the dissolution medium were determined by HPLC, which comprised of Waters Alliance 2695 (Waters, Milford, MA, USA) coupled with the Waters photodiode array detector 2996 (Waters). Sildenafil was separated with a Luna^®^ 3 μm C18(2) column, (150 × 3 mm, i.d.; Phenomenex, Torrance, CA, USA) with an isocratic solvent system consisting of acetonitrile and water at 30:70, 90:10, and 60:40 (*v*/*v*) as the mobile phase for the dissolution medium at pH 1.2, 4.5, and 6.8, respectively. The flow rate was 0.3 mL/min and the total run time was 4.0 min. The column oven temperature was set at 30 °C. The sample injection volume was 10 μL and sildenafil was detected at 230 nm. Working standard solutions of sildenafil for HPLC analyses were prepared in each dissolution medium and diluted with acetonitrile at concentrations of 0.5, 1, 5, 10, 20, 50, and 100 μg/mL.

#### 2.5.2. LC-MS/MS

Sildenafil concentrations in Beagle dog plasma were determined by an LC-MS/MS method. The LC-MS/MS system comprised of an Agilent 6430 triple-quadrupole mass spectrometer coupled with an Agilent 1200 HPLC (Agilent Technologies, Santa Clara, CA, USA). Plasma samples were prepared by a protein precipitation method using methanol. Sildenafil was separated with a Zorbax SB-Aq column (2.1 × 150 mm, i.d., 5 μm; Agilent Technologies) with an isocratic solvent system consisting of 0.1% formic acid in water and methanol (50:50, *v*/*v*) as the mobile phase and a flow rate of 0.3 mL/min. The column oven temperature was 30 °C and the total run time was 6.5 min. The mass spectrometer was operated using electron spray ionization in the positive ion mode with mass transitions of m/z 475.2 → 58.2 for sildenafil and m/z 390.2 → 268.1 for tadalafil (IS). The assay was validated using the matrix-matched quality control (QC) samples. The lower limit of quantification (LLOQ) was 5 ng/mL and the intra- and inter-day accuracy and precision ranged from 97.2% to 107.0% and within 7.4%, respectively.

### 2.6. POP-PK IVIVC Model

#### 2.6.1. Non-Compartmental Analysis

The non-compartmental pharmacokinetic parameters were determined by using Phoenix^®^ WinNonlin^®^ (Certara, L.P., Princeton, NJ, USA). The non-compartmental pharmacokinetic parameters included terminal half-life (*t*_1/2_), area under the plasma concentration versus time curve from time zero to the last observation time point (AUC_all_) and to infinity (AUC_inf_), apparent volume of distribution (*V*_z_/F), and apparent systemic clearance (CL/F). The maximum plasma concentration (*C*_max_) and the time to reach *C*_max_ (*T*_max_) were obtained directly from the observed data. Relative bioavailability (BA) of the different sildenafil SR tablets was estimated by the ratio of the dose-normalized AUC_inf_ of a specific SR tablet compared and that of the IR tablet. 

#### 2.6.2. In Vitro Dissolution Modeling

Since drug dissolution was not completed at a higher pH, the in vitro dissolution profiles of IR and three different SR tablets determined at pH 1.2 medium were used for the in vitro dissolution modeling. The in vitro dissolution profiles were characterized by the Michaelis–Menten equation. The differential equation for the amount of sildenafil in the tablet (X_Tablet, in vitro_) was:

(1) $$\frac{{\mathrm{dX}}_{\mathrm{Tablet},\mathrm{in}\mathrm{vitro}}}{\mathrm{dt}}=-\frac{V_{\max,\mathrm{in}\mathrm{vitro}}}{{\mathrm{AM}}_{50,\mathrm{in}\mathrm{vitro}}+X_{\mathrm{Tablet},\mathrm{in}\mathrm{vitro}}}\cdot X_{\mathrm{Tablet},\mathrm{in}\mathrm{vitro}}$$

where, V_max, in vitro_ represents the maximum rate of drug release in the dissolution tester, and AM_50, in vitro_ is the amount of drug at which the dissolution rate is half of V_max, in vitro_. Since the dissolution profiles were more sensitive to V_max, in vitro_ than to AM_50, in vitro_, the estimated V_max, in vitro_ was used as a representing dissolution parameter to compare dissolution rates of different SR tablets.

#### 2.6.3. Population Pharmacokinetic Modeling 

**Figure 1:**
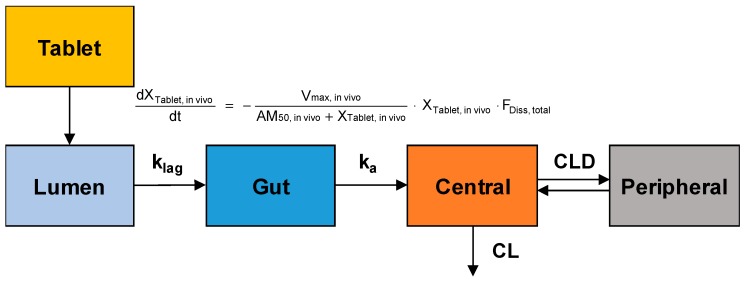
A structural model for the dissolution, absorption, and disposition of sildenafil after oral administration of sildenafil tablets. X_Tablet, in vivo_, the amount of drug in the tablet; V_max, in vivo_, in vivo maximum dissolution rate; AM_50, in vivo_, the amount of drug at which the dissolution rate is half of V_max, in vivo_; F_Diss, total_, the dissolved fraction that can change over time; k_lag_, the first order rate constant representing the transfer of the dissolved sildenafil from the lumen to the gut compartment; k_a_, first-order absorption rate constant; CL, systemic clearance; CLD, distribution clearance.

The obtained plasma concentration versus time data after oral administration of sildenafil tablets were fitted to the population-pharmacokinetic (POP-PK) model to estimate the in vivo dissolution profiles of sildenafil from IR and SR tablets. The model structure for the POP-PK modeling of sildenafil is shown in Figure 1.

The in vivo dissolution of sildenafil from the IR and SR tablets was also described by the Michaelis–Menten kinetics. The differential equation for the amount of drug in the tablet (X_Tablet, in vivo_) was written as follows:

(2) $$\frac{{\mathrm{dX}}_{\mathrm{Tablet},\mathrm{in}\mathrm{vivo}}}{\mathrm{dt}}=-\frac{V_{\max,\mathrm{in}\mathrm{vitro}}}{{\mathrm{AM}}_{50,\mathrm{in}\mathrm{vitro}}+X_{\mathrm{Tablet},\mathrm{in}\mathrm{vitro}}}\cdot X_{\mathrm{Tablet},\mathrm{in}\mathrm{vivo}}\cdot F_{\mathrm{Diss},\mathrm{total}}$$

where, V_max, in vivo_ is the in vivo maximum drug release rate in the gastrointestinal tract, and AM_50, in vivo_ is the amount of drug at which the dissolution rate is half of V_max, in vivo_. To correct the influence of the different doses of sildenafil in the IR and SR formulations, the amount of sildenafil in the tablet was normalized by the dose and the dose-normalized V_max, in vivo_ and AM_50, in vivo_ were estimated by the modeling. Since the dissolution of sildenafil is highly pH-dependent, the dissolved fraction (F_Diss, total_) which can change over time as the drug passes through the gastrointestinal tract was incorporated. F_Diss, total_ is described by using the following Hill-type equations:

(3) $$F_{\mathrm{Diss},\mathrm{total}}=F_{\mathrm{Diss},\mathrm{stomach}}+F_{\mathrm{Diss},\mathrm{intestine}}$$

(4) $$F_{\mathrm{Diss},\mathrm{stomach}}=\left\{1-I_{\max}\cdot \frac{{\mathrm{Time}}^{{\mathrm{Hill}}_{\mathrm{stomach}}}}{T_{\mathrm{GET}}{}^{{\mathrm{Hill}}_{{}_{\mathrm{stomach}}}}+{\mathrm{Time}}^{{\mathrm{Hill}}_{\mathrm{stomach}}}}\right\}$$

(5) $$F_{\mathrm{Diss},\mathrm{intestine}}={\mathrm{Diss}}_{\max}\cdot \left\{\frac{{\mathrm{Time}}^{{\mathrm{Hill}}_{\mathrm{intestine}}}}{T_{\mathrm{trans}1}{}^{{\mathrm{Hill}}_{\mathrm{intestine}}}+{\mathrm{Time}}^{{\mathrm{Hill}}_{\mathrm{intestine}}}}\right\}\cdot \left\{1-\frac{{\mathrm{Time}}^{{\mathrm{Hill}}_{\mathrm{intestine}}}}{T_{\mathrm{trans}2}{}^{{\mathrm{Hill}}_{\mathrm{intestine}}}+{\mathrm{Time}}^{{\mathrm{Hill}}_{\mathrm{intestine}}}}\right\}$$

(6) $$T_{\mathrm{trans}1}=T_{\mathrm{GET}}+T_{\mathrm{ITT}}$$

(7) $$T_{\mathrm{trans}2}=T_{\mathrm{GET}}+T_{\mathrm{ITT}}+T_{\mathrm{CTT}}$$

where, F_Diss, stomach_ and F_Diss, intestine_ are the dissolved fractions which modulate the dissolution rate of sildenafil in the stomach and intestine, respectively. I_max_ is the maximum decreased fraction of F_Diss, stomach_, Time is the accumulated time after drug administration, and T_GET_ refers to the time associated with a half-maximal decrease of F_Diss, stomach_ during gastrointestinal pH increase from the stomach to the intestine, i.e., gastric emptying. Diss_max_ is the maximum increased fraction of F_Diss, intestine_. T_trans1_ and T_trans2_ are the times associated with the half-maximal increase and decrease of F_Diss, intestine_ respectively, which occurs due to the pH changes along the gastrointestinal tract during the intestinal migration of sildenafil. Hill_stomach_ and Hill_intestine_ are Hill coefficients to determine the steepness of changes in F_Diss, stomach_ and F_Diss, intestine_, respectively. T_ITT_ and T_CTT_ represent intestinal transit time and colon transit time, respectively. Consequently, F_Diss, total_, the sum of F_Diss, stomach_, and F_Diss, intestine_, changes over time as the drug pass through the gastrointestinal tract.

Following dissolution, the transfer of the dissolved sildenafil from the lumen to the gut compartment and absorption from the gut compartment to the central compartment were described by first-order processes with the rate constant of k_lag_ and k_a_, respectively. The differential equations for the dissolved amount of sildenafil in the lumen and gut compartments were given by:

(8) $$\frac{{\mathrm{dX}}_{\mathrm{Lumen}}}{\mathrm{dt}}=\frac{V_{\max,\mathrm{in}\mathrm{vitro}}}{{\mathrm{AM}}_{50,\mathrm{in}\mathrm{vitro}}+X_{\mathrm{Tablet},\mathrm{in}\mathrm{vitro}}}\cdot X_{\mathrm{Tablet},\mathrm{in}\mathrm{vivo}}\cdot F_{\mathrm{Diss},\mathrm{in}\mathrm{vivo}}-k_{\mathrm{lag}}\cdot X_{\mathrm{Lumen}}$$

(9) $$\frac{{\mathrm{dX}}_{\mathrm{Gut}}}{\mathrm{dt}}=k_{\mathrm{lag}}\cdot X_{\mathrm{Lumen}}-k_{a}\cdot X_{\mathrm{Gut}}$$

The systemic disposition of sildenafil was described by a two-compartment model. The amount of sildenafil in the central compartment (X_1_) was assumed to be distributed to the peripheral compartment (X_2_) and eliminated from the central compartment. The differential equations for the amount of sildenafil in the central and peripheral compartments were:

(10) $$\frac{{\mathrm{dX}}_{1}}{\mathrm{dt}}=k_{a}\cdot X_{\mathrm{Gut}}-\mathrm{CLD}\cdot C_{1}+\mathrm{CLD}\cdot C_{2}-\mathrm{CL}\cdot C_{1}$$

(11) $$\frac{{\mathrm{dX}}_{2}}{\mathrm{dt}}=\mathrm{CLD}\cdot C_{1}-\mathrm{CLD}\cdot C_{2}$$

where, C_1_ and C_2_ represent sildenafil concentrations in the respective compartments, and CLD and CL are the distribution clearances to the peripheral compartment and the systemic clearance, respectively. 

The observed plasma concentration-time data following oral administration of IR and three SR tablets were simultaneously fitted to the POP-PK model using the Monte Carlo Parametric Expectation Maximization (MC-PEM) algorithm in the parallelized S-ADAPT software (version 1.57). An importance sampling MC-PEM method (pmethod = 4 in S-ADAPT) was used for population parameter estimation. Between-subject variability (BSV) was estimated using an exponential parameter variability model. The predictive performance of the POP-PK model was evaluated by visual predictive checks. Simulations were performed by using the Berkeley Madonna software (version 8.3.18). 

#### 2.6.4. Correlation of In Vitro and In Vivo Dissolution

The estimated in vitro and in vivo dissolution rates, V_max, in vitro_ and V_max, in vivo_ for the IR and three SR tablets were correlated via regression analysis using SigmaPlot (version 12.5, Systat Software, Inc., CA, USA). The coefficient of determination (*r*^2^) was calculated to assess the goodness-of-fit. The equation for correlation was then applied to the developed POP-PK model to convert in vitro dissolution profiles to in vivo ones.

### 2.7. Validation of the POP-PK IVIVC Model 

Based on the final POP-PK IVIVC model, the individual plasma concentration versus time profile of sildenafil was predicted by Monte Carlo simulations using Berkeley Madonna (version 8.3.18). The predictive performance was evaluated by comparing the predicted and observed values of the mean C_max_ and AUC_all_. The absolute percentage of prediction error (%PE) was calculated as:

(12) $$\%\mathrm{PE}=\frac{\left|\mathrm{Observed}-\mathrm{Predicted}\right|}{\mathrm{Observed}}\times 100$$

## 3. Results

### 3.1. In Vitro Dissolution of Sildenafil from SR Tablets

**Figure 2:**
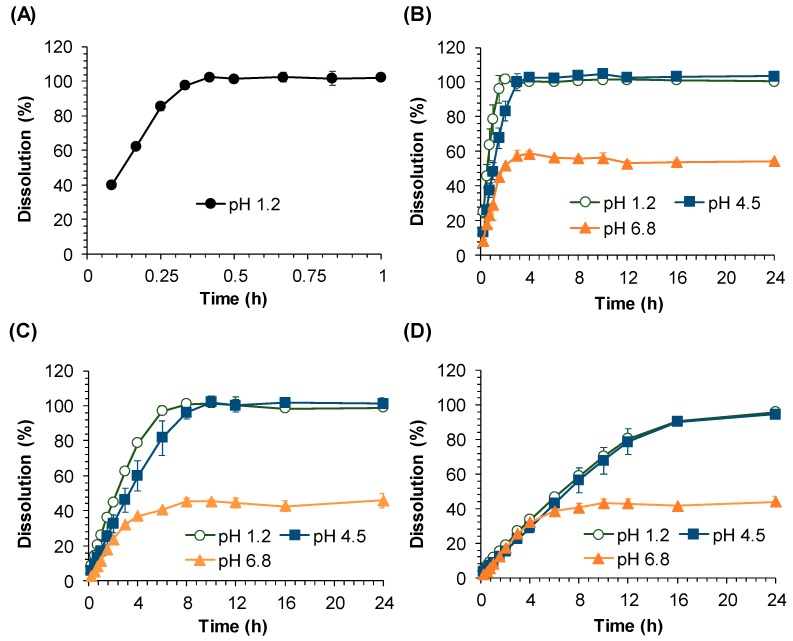
In vitro dissolution profiles of sildenafil from (**A**) Immediate Release (IR) tablet at pH 1.2 medium, (**B**) Sustained Release (SR) SR_fast_, (**C**) SR_medium_, and (**D**) SR_slow_ tablets at pH 1.2, 4.5, and 6.8 media.

Figure 2 shows the in vitro dissolution profiles of sildenafil IR tablets at pH 1.2 and SR tablets at pH 1.2, 4.5, and 6.8. While sildenafil release from IR tablets was completed within 0.4 h at pH 1.2 (Figure 2A), drug releases from SR tablets were significantly delayed as their HPMC compositions increased (Figure 2B–D). Among the SR tablets, sildenafil release was the fastest from SR_fast_ followed by SR_medium_ and SR_slow_ tablets regardless of the pH of the medium. For each SR tablet, the dissolution was significantly pH-dependent. While the dissolution profiles from SR tablets at pH 1.2 and pH 4.5 were comparable, the dissolution of sildenafil at pH 6.8 was significantly slower than those at pH 1.2 and 4.5 media and was not completed after 24 h. At pH 6.8, a maximum drug release of 57.6% was achieved after 3 h from SR_fast_, and a maximum drug release of approximately 40.0% was achieved at 6–8 h from SR_medium_ and SR_slow_, no further drug releases were observed thereafter. 

### 3.2. In Vivo Pharmacokinetics of Sildenafil 

**Figure 3:**
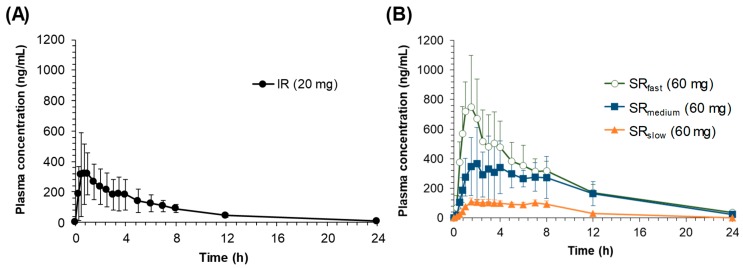
Average plasma concentration versus time profiles of sildenafil following oral administration of sildenafil (A) IR and (B) SR tablets (*n* = 3, each).

The individual plasma concentration-time profiles of sildenafil following oral administration of IR (sildenafil 20 mg) and SR tablets (sildenafil 60 mg) in Beagle dogs are shown in Figure 3. The non-compartmental pharmacokinetic parameters of sildenafil are summarized in Table 2. Upon oral administration of the sildenafil IR tablet, the plasma concentrations of sildenafil rapidly increased and reached its peak concentration (*C*_max_) within 1 h. Then, the plasma concentrations declined with the average elimination half-life (*t*_1/2_) of 4.3 ± 0.7 h. Following the oral administration of the SR tablets, a longer *T*_max_ was observed compared to the IR tablet, which was further prolonged as their dissolution rates decreased. Although an extended absorption was observed, the *C*_max_ and AUC were reduced following the oral administration of SR tablets. The decreases in *C*_max_ and AUC were most prominent for the SR_slow_ formulation, followed by SR_medium_ and SR_fast_, resulting in the average relative bioavailability (%) of 18.9%, 72.8%, and 101.1%, respectively.

Interestingly, second peaks in plasma concentrations were also observed in the individual plasma concentration-time profiles. The second peaks were more distinct following oral administrations of the slower release formulations, i.e., SR_slow_ and SR_medium_, while they were less apparent and appeared more like shoulder peaks in the plasma concentration-time profiles following oral administrations of SR_fast_ and IR.

### 3.3. Establishment of POP-PK IVIVC Model 

#### 3.3.1. In Vitro Dissolution Model 

Since the dissolution from SR tablets was not completed at higher pH (Figure 2), the in vitro dissolution data obtained at pH 1.2 and 4.5 medium were used. To fit the in vitro dissolution profiles at pH 1.2 and 4.5, zero-order, first-order, and Michaelis–Menten kinetics have been tested and the best fitting was obtained from the Michaelis–Menten equation at pH 1.2. Thus, the in vitro drug release profiles of sildenafil from IR and SR tablets obtained at pH 1.2 were fitted to a Michaelis–Menten kinetic model (Equation (1)) and used for the development of the IVIVC model. The developed in vitro dissolution model adequately described overall drug release profiles (Supplementary Figure S1). The estimated maximum drug release rates, V_max, in vitro_ and the Michaelis–Menten constant, AM_50, in vitro_, are summarized in Table 3. The estimated V_max, in vitro_ was the highest for IR followed by SR_fast_, SR_medium_, and SR_slow_.

#### 3.3.2. Estimation of the In Vivo Dissolution Profile 

To describe the plasma concentration versus time profiles of sildenafil and extract the in vivo dissolution profiles, the data obtained from pharmacokinetic studies were simultaneously fitted to the POP-PK model (Figure 1). The developed POP-PK model adequately described the overall plasma concentration-time profiles of sildenafil, as indicated by the plots of the observed and fitted values (Supplementary Figure S2). The final parameter estimates of the POP-PK model are presented in Table 4.

**Figure 4:**
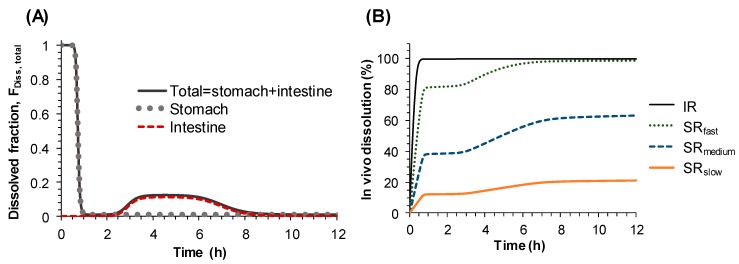
Model-estimated (**A**) dissolved fraction (F_Diss, total_) and (**B**) in vivo dissolution of sildenafil (%) from IR and SR tablets.

In this model, in vivo dissolution and systemic absorption were separately described. The in vivo dissolution process was modeled by multiplying the overall dissolution rate for each SR tablet described by the Michaelis–Menten kinetics and the time-dependent dissolved fraction (F_Diss, total_) described by Hill equations. While all other parameters to describe the in vivo dissolution were shared among all groups, V_max, in vivo_ was separately estimated for IR, SR_fast_, SR_medium_, and SR_slow_ tablets. In Figure 4, the estimated F_Diss, total_, which is the sum of F_Diss, stomach_ and F_Diss, intestine_, and in vivo dissolution (%) of the four formulations are presented. Following oral administration of sildenafil formulations, the initial F_Diss, total_ was estimated to be 100% until approximately 30 min and decreased to the minimum fraction of 0.8% at 1 h. At 2 h post dosing, F_Diss, total_ moderately increased and remained above 10% within 3.2–6.3 h after drug administration due to the slight increase of F_Diss, intestine_ and gradually decreased to minimum thereafter. As the F_Diss, total_ reached the minimum again at around 10 h after dosing, the percentage of sildenafil dissolved in SR_medium_ and SR_slow_ was limited to 63% and 21% at 12 h respectively, while the in vivo release from IR and SR_fast_ formulations were almost completed (Figure 4B).

#### 3.3.3. Correlation of In Vitro and In Vivo Dissolution 

**Figure 5:**
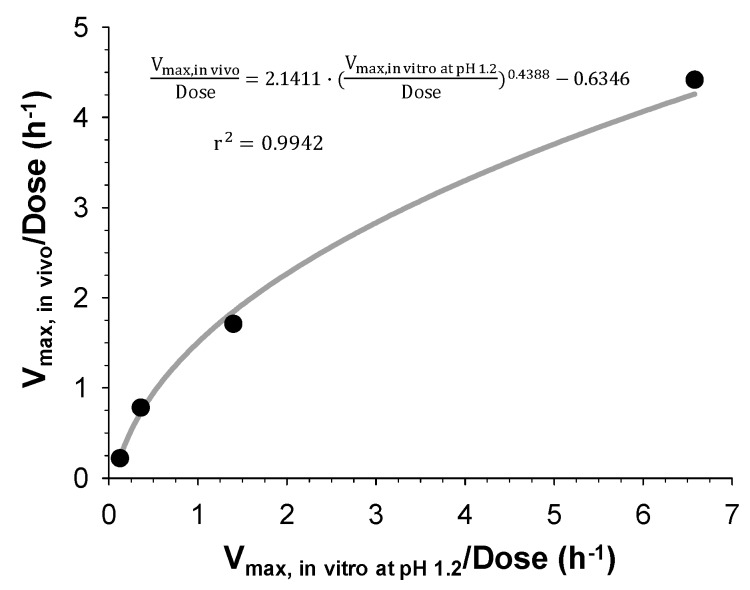
Correlation between the V_max_ estimates for in vitro and in vivo dissolution by a power regression.

It was noticed that the dissolution rates determined in vitro were slower than those determined in vivo. Thus, the interconversion between in vitro and in vivo dissolution profiles was necessary. Although a one-to-one correlation may be preferred, the in vitro dissolution obtained from experiments and that estimated by the POP-PK model could not be superimposable, which may be due to the complexity of the gastrointestinal environment (e.g., transit time and pH). Therefore, the in vitro and in vivo dissolution model estimates, i.e., V_max, in vitro_ and V_max, in vivo_ of the IR and the three SR tablets were correlated, as previously reported [5,6]. Since the obtained dose-normalized V_max, in vitro_ and V_max, in vivo_ ranged widely from 0.538 to 6.58 and from 0.219 to 4.42, the power equation was found to be the best model (Equation (13)). Figure 5 shows the correlation between the V_max_ estimates for in vitro and in vivo.

(13) $$V_{\max,\mathrm{in}\mathrm{vivo}}/\mathrm{Dose}=2.1411\cdot {{(V}_{\max,\mathrm{in}\mathrm{vitro}\mathrm{at}\mathrm{pH}1.2}/\mathrm{Dose})}^{0.4388}-0.6346$$

#### 3.3.4. Validation of the POP-PK IVIVC Model

**Figure 6:**
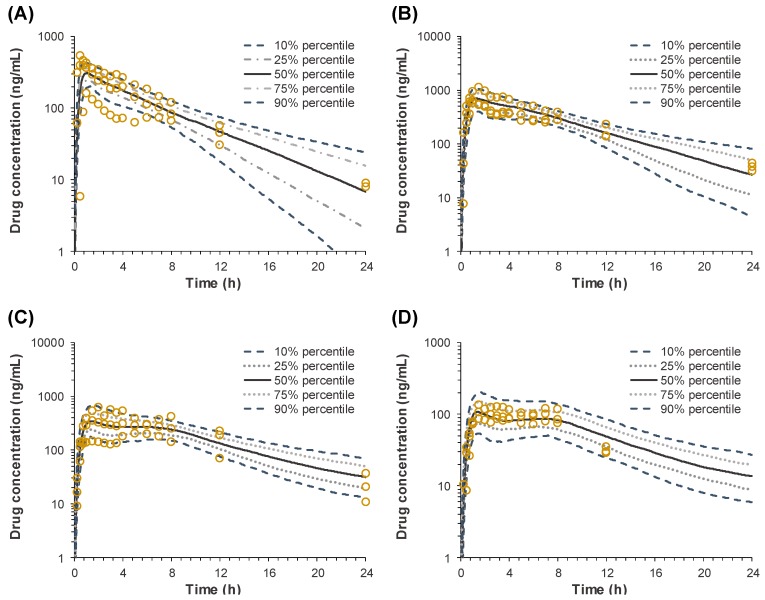
Estimation of the in vivo plasma concentration versus time profiles after oral administration of sildenafil (**A**) IR, (**B**) SR_fast_, (**C**) SR_medium_, and (**D**) SR_slow_ tablets by the in vitro-in vivo correlation (IVIVC) model.

Finally, the predictability of the developed POP-PK IVIVC model was evaluated by Monte Carlo simulations. The predicted plasma concentration versus time profiles of sildenafil following oral administration of the IR and SR tablets from the in vitro dissolution profiles are presented in Figure 6. The visual predictive checks for all of the plasma concentration versus time profiles showed reasonable model predictability for the IR and SR tablets. The predicted *C*_max_ and AUC_all_ in comparison with the observed values, and their respective absolute percentages of prediction error (%PE), are shown in Table 5. The predicted *C*_max_ and AUC_all_ were close to the observed values with the %PE of 4.0–9.8% and 0.1–7.7% respectively, and the mean %PE of 7.2% ± 2.4% for *C*_max_ and 3.5% ± 3.4% for AUC, satisfying the FDA criteria [1]. 

## 4. Discussion

Oral absorption behavior of a drug whose solubility is heavily dependent on pH often exhibits unusual patterns due to its complex dissolution profiles in the gastrointestinal tract. Since such a complex pH-dependent dissolution is hard to be predicted by using conventional IVIVC methods, the demand for a new IVIVC method that can describe the complex dissolution and absorption process in the gastrointestinal tract is growing. In the present study, we have established a level-A IVIVC model applicable to drugs that exhibit complex pharmacokinetics due to the complex dissolution process associated with pH-dependent solubility by using sildenafil as a model drug. 

The in vitro dissolution study clearly suggests the highly pH dependent dissolution of sildenafil from SR formulations. Our data showed that while sildenafil releases from SR tablets were completed at pH 1.2 and 4.5, the drug release at pH 6.8 was incomplete and only 60% of the dissolution was achieved. The pH-dependent dissolution of sildenafil may be contributed to its variable solubility depending on pH [11,12,13]. Sildenafil was reported to meet the criteria of “high solubility” at a pH of 1.2 and 4.5 according to WHO guidelines [11,14]. However, with the pH increase, the solubility sharply dropped from pH 4 to 6 and the lowest solubility was observed at around pH 7 and 8 [11,12]. These pH-dependent properties of sildenafil dissolution indicate that the in vivo dissolution, as well as the absorption process, of sildenafil SR formulations may be significantly affected by the gastrointestinal pH. The gastrointestinal pH is reported to be 3.5 in the posterior stomach, increasing up to the highest pH of pH = 7.5 at the end of the small intestine, and decreasing to pH = 6.5 in the colon in dogs [15]. Although the pH difference between the small intestine (pH = 7.5) and the colon (pH = 6.5) is small, the in vivo dissolution of sildenafil within this pH range may be significantly affected, as suggested by the five-fold increase of solubility as pH decreases from 7 to 6 [11]. The complete dissolution of sildenafil at pH 1.2 and 4.5 and the incomplete dissolution at pH 6.8 observed in this study are in good agreement with its reported solubility at these pH values. Thus, it can be assumed that sildenafil dissolution may be complete only at gastric pH and the absorption of sildenafil may be most efficient in the stomach, followed by that in the large intestine and small intestine where sildenafil is poorly soluble. 

The pH-dependent dissolution properties of sildenafil tablets might have led to the distinctive in vivo pharmacokinetic characteristics observed following the oral administration of IR and SR tablets. In the in vivo pharmacokinetics studies, the lower *C*_max_ and AUC values were observed for the formulations with slower drug release rates. While the oral bioavailability for SR_fast_ was comparable with that for the IR tablet, the average relative bioavailability for SR_medium_ and SR_slow_ was significantly decreased to 72.8% and 18.9%, respectively (Table 2). The reduced bioavailability with the dissolution rate decrease has been observed for drugs that exhibit site-specific absorption [5,6]. Since the drug is exclusively absorbed in the upper part of the gastrointestinal tract, the slowly released drug from the SR formulations in the intestine, i.e., after passing the main absorption site, may not be absorbed, leading to the lower bioavailability. In the case of sildenafil, the upper part of the gastrointestinal tract with the lower pH may provide the main absorption site of sildenafil in the gastrointestinal tract. 

Another interesting feature observed in the in vivo pharmacokinetic study was the secondary peaks in the plasma concentration-time profiles following the oral administration of sildenafil SR tablets. The secondary peaks were also observed after oral administration of sildenafil at a higher dose in healthy dogs [16]. However, the secondary peaks in the plasma concentration could not be easily observed either in Beagle dogs [17] or in humans [18,19] following oral administration of the sildenafil IR tablet, which is consistent with the present results. As shown in Figure 3, the secondary peaks are less apparent after oral administration of IR, whereas they are more distinct after oral administrations of the slower release formulations. It is generally perceived that the secondary peaks in plasma concentrations could result from enterohepatic circulation or the presence of site-specific drug absorption and dissolution [6,20]. Enterohepatic circulation is a phenomenon in which drugs absorbed to the systemic circulation are excreted into the bile and reabsorbed, resulting in the secondary peaks in the plasma concentration-time profile [20]. Thus, the size of the second peak resulting from enterohepatic circulation is generally proportional to the first peak, because the second peak is originated from the initial absorption of a drug, which is reflected in the first peak, followed by excretion into bile and reabsorption. In this study, however, although the size of the first peak decreased as the drug dissolution rate decreased, the relative size of the second peak compared to the first one increased. The average ratio of the second peak to the first one (*C*_max, second_/*C*_max, first_) was 0.51 and 0.56 in IR and SR_fast_ respectively, but increased to 0.94 and 0.92 in SR_medium_ and SR_slow_, respectively. The increase of the relative size of the second peaks may be because the slower the drug release rate is, the more of the unabsorbed drug is left remaining in the gastrointestinal tract, which was dissolved at a slightly lower pH in the colon and reflected in the secondary peaks. Thus, the secondary peaks of sildenafil in the plasma concentration-time profiles may be originated from the direct absorption of the remaining drug in the lower part of the gastrointestinal tract, i.e., colon, not from enterohepatic circulation. Furthermore, even though its metabolites are reported to undergo enterohepatic circulation, sildenafil itself is not excreted into bile [21], suggesting that the secondary peaks of sildenafil may not be associated with enterohepatic circulation. We also applied several reported PK model structures of enterohepatic circulation [22,23,24] to examine if they could describe the second peak of sildenafil observed in our study. However, the observed in vivo data could not be fitted by those models (data are not shown).

Sildenafil citrate is known to be a BCS class II drug which has low solubility and high permeability. Several studies have conducted in vitro permeability assays utilizing Caco-2, MDCK, and PAMPA, from which they have confirmed the high permeability of sildenafil [11]. From an in situ intestinal perfusion study in rabbits, it was found that sildenafil could be absorbed throughout the gastrointestinal tract [25]. Thus, the absorption of sildenafil may be mainly dependent on its dissolution rate in the gastrointestinal tract. As indicated by its variable solubility depending on pH, the dissolution rate of sildenafil would be the highest in the stomach, the lowest in the small intestine, and slightly higher again in the colon as SR formulations move along the gastrointestinal tract. Therefore, the second peak of sildenafil may be attributed to the changes in the dissolution rate depending on the regional pH of the gastrointestinal tract. While the first peak in the plasma concentrations is due to the dissolved drug in the stomach, the second peak may be the result of the drug that dissolved in the colon. 

Therefore, instead of describing the second peak in the plasma concentration-time profile by systemic disposition, i.e., enterohepatic circulation, it was described by the changes of dissolution rate from sildenafil tablets along the gastrointestinal tract. To represent the changes of the dissolution rate of sildenafil along the gastrointestinal tract, the dissolved fraction that can change over time (F_Diss, total_) was incorporated into the Michaelis–Menten equation (Equation (2)). The time-dependent F_Diss, total_, which was modeled by Hill type equations (Equations (3)–(7)), enabled us to describe the increase and decrease of the dissolution rate of sildenafil formulations along the gastrointestinal tract, i.e., the high dissolution rate in the stomach and low dissolution in the small intestine, followed by moderate dissolution in the colon. The final model adequately described the secondary peaks in the plasma concentration-time profiles obtained after oral administration of sildenafil IR and SR tablets and provided reasonable parameter estimates. The times for 50% of gastric emptying, intestinal transit, and colon transit were estimated to be 0.73, 2.18, and 4.1 h, respectively. The physiological transit time for stomach has been reported to be 96 min, 110 min for the small intestine, and 770 min for the whole gut [20]. The dissolved fraction over time (F_Diss, total_) was predicted to be almost 100% in the stomach, rapidly decreasing to nearly zero as the tablet passes through the small intestine, then slightly increasing again and remaining so during the drug transit in the large intestine, and finally to reach zero at the end of the gastrointestinal tract (Figure 4A). Based on the POP-PK model, the complex in vivo dissolution profiles of sildenafil from the IR and SR tablets could be estimated (Figure 4B).

Finally, IVIVC has been established by correlating the estimated in vitro and in vivo dissolution parameters, V_max, in vitro_ and V_max, in vivo_, for the IR and three SR tablets with different dissolution rates. Both V_max, in vitro_ and V_max, in vivo_ were the highest for the IR tablet and decreased with the increasing HPMC amount in the SR tablets. The linear and power regression were tested to find the best correlation between the obtained in vitro and in vivo V_max_. As a result, the power regression showed the higher correlation coefficients (Figure 5) and the IVIVC model was developed based on the power regression. The final IVIVC model allowed for the prediction of the plasma concentration-time profiles of sildenafil from their in vitro release profile. The predicted plasma concentration-time profiles of sildenafil SR formulations were in good agreement with the observed data. The predictability of the developed IVIVC model was validated by Monte Carlo simulations. The predictive performance of the model for C_max_ and AUC compared to the observed values satisfied the FDA criteria, indicating the establishment of a level-A IVIVC for sildenafil [1]. 

Although this study determined the in vitro dissolution characteristics of sildenafil formulations at three different pH levels, the present in vitro dissolution study was intended to evaluate the pH-dependency of the sildenafil dissolution, not to mimic the in vivo gastrointestinal environment. It might also be possible to determine the in vitro dissolution at various pH levels that exactly correspond to the in vivo pH of the gastrointestinal segments and then establish a one-to-one correlation between the in vitro and in vivo dissolution at physiological pH. However, since this study focused on establishing an IVIVC for drugs with pH-dependent dissolution, we selected the in vitro dissolution rate at pH 1.2 (V_max, in vitro_) as a representative in vitro dissolution parameter after characterizing the in vitro dissolution at different pH and correlated it with the in vivo dissolution parameter, V_max, in vivo_, to establish IVIVC. Although this approach may be empirical, the acceptable predictability of the developed IVIVC has been demonstrated, and the in vivo dissolution profile in the gastrointestinal tract depending on the physiological pH was successfully estimated based on the in vivo data for the IR and three SR tablets. 

## 5. Conclusions

In summary, a level-A IVIVC has been established for sildenafil using POP-PK modeling that accounted for the pH-dependent dissolution by applying the dissolution fraction parameter (F_Diss, total_) that can change over time and modulate the dissolution rate in the gastrointestinal tract. A power regression was used to correlate the in vitro and in vivo dissolution profiles of sildenafil IR and three SR tablets. Using the in vitro dissolution profile as input data, the final IVIVC model could successfully predict the complex in vivo pharmacokinetic profiles of sildenafil, which included second peaks and reduced bioavailability as the dissolution rate decreased. We believe that this new approach holds great promise for the establishment of IVIVCs for drugs with poor predictability by conventional IVIVC methods due to complex dissolution along the gastrointestinal tract and provides useful information for improving the success rate of formulation development. 

## Figures and Tables

**Table 1:** Compositions (*w*/*w* %) of sildenafil formulations.

Substances	IR	SR_fast_	SR_medium_	SR_slow_
Sildenafil (mg per tablet)	20	60	60	60
Sildenafil citrate	5.6	16.9	16.9	16.9
Lactose	74.2	72.9	52.9	22.9
HPMC2208-100 cps	-	10.0	30.0	60.0
Polyvinylpyrrolidone K30	10.0	-	-	-
Na CMC	10.0	-	-	-
Mg stearate	0.2	0.2	0.2	0.2

**Table 2:** Pharmacokinetic parameters of sildenafil obtained after oral administration of sildenafil IR and SR tablets in Beagle dogs (*n* = 3, each).

Parameters	IR	SR_fast_	SR_medium_	SR_slow_
Dose (mg)	20	60	60	60
*t*_1/2_ (h)	4.3 ± 0.7	5.3 ± 0.8	4.6 ± 1.1	2.8 ± 0.7
*T*_max_ (h)	0.8 ± 0.3	1.0 ± 0.4	2.7 ± 2.1	5.0 ± 2.8
*C*_max_ (ng/mL)	366.5 ± 191.5	831 ± 285.2	410.3 ± 211.9	124.9 ± 4.6
AUC_all_ (ng·h/mL)	1913.2 ± 1,019.3	5885 ± 1,914.2	4221.3 ± 1,920.3	1009.0 ± 31.3
AUC_inf_ (ng·h/mL)	2007.1 ± 956.4	6087.4 ± 1,773.2	4381.7 ± 1,960.0	1140.8 ± 76.6
V_z_/F (L)	73.0 ± 35.0	81.0 ± 28.5	107.2 ± 58.2	208.0 ± 35.8
CL/F (mL/min)	202.3 ± 117.1	172.6 ± 43.1	272.4 ± 151.0	878.6 ± 59.0
Relative BA (%)	-	101.1 ± 29.4	72.8 ± 32.6	18.9 ± 1.3

**Table 3:** In vitro dissolution parameter estimates.

Parameter	Symbols (Unit)	Population Mean (BSV*)
Amount of sildenafil in the X_tablet, in vitro_ associated with the 1/2 V_max, in vitro_	AM_50, in vitro_/dose (no unit)	0.395 (0.0316)
V_max, in vitro_ for IR tablet	V_max, in vitro, IR_/dose (1/h)	6.58 (0.0283)
V_max, in vitro_ for SR_fast_ tablet	V_max, in vitro, SRfast_/dose (1/h)	1.40 (0.147)
V_max, in vitro_ for SR_medium_ tablet	V_max, in vitro, SRmedium_/dose (1/h)	0.358 (0.0247)
V_max, in vitro_ for SR_slow_ tablet	V_max, in vitro, SRslow_/dose (1/h)	0.124 (0.0333)

*, between subject variability.

**Table 4:** Population pharmacokinetic parameter estimates of sildenafil.

Parameter	Symbol (Unit)	Population Mean (BSV)
Volume of distribution of the central compartment	V1 (L)	22 (0.157)
Volume of distribution of the peripheral compartment	V2 (L)	31 (0.751)
Systemic clearance	CL (L/h)	49.2 (0.379)
Distribution clearance	CLD (L/h)	9.97 (0.2)
Rate constant for drug transfer from lumen to gut	k_lag_ (1/h)	3.13 (0.497)
Rate constant for absorption from gut	k_a_ (1/h)	4.37 (0.952)
Time for half-maximal decrease of F_Diss, stomach_	T_GET_ (h)	0.73 (0.385)
Maximum fraction of F_Diss, stomach_ decrease	I_max_	0.992 (0.0027)
Hill coefficient for the decrease of F_Diss, stomach_	Hill_stomach_	16.7 (0.0316)
Intestinal transit time	T_ITT_ (h)	2.18 (0.434)
Colon transit time	T_CTT_ (h)	4.1 (0.11)
Maximum fraction of F_Diss, intestine_ increase	Diss_max_	0.115 (0.359)
Hill coefficient for the decrease of F_Diss, intestine_	Hill_intestine_	13.1 (0.0316)
Amount of sildenafil in the X_solid_ at 1/2 V_max, in vivo_	AM_50, in vivo_/dose	0.315 (0.105)
V_max, in vivo_ for IR tablet	V_max, in vivo, IR_/dose (1/h)	4.42 (0.416)
V_max, in vivo_ for SR_fast_ tablet	V_max, in vivo, SRfast_/dose (1/h)	1.71 (0.145)
V_max, in vivo_ for SR_medium_ tablet	V_max, in vivo, SRmedium_/dose (1/h)	0.781 (0.295)
V_max, in vivo_ for SR_slow_ tablet	V_max, in vivo, SRslow_/dose (1/h)	0.219 (0.255)

**Table 5:** Prediction error (%PE) for *C*_max_ and area under the plasma concentration-time curve (AUC) of sildenafil obtained from IVIVC modeling.

Formulation	*C*_max_ (ng/mL)			AUC (ng·h/mL)		
	Observed	Predicted	PE (%)	Observed	Predicted	PE (%)
IR	366.5 ± 191.5	338.7	7.6	1913.2 ± 1019.3	1942.2	1.5
SR_fast_	831 ± 285.2	797.4	4.0	5,885 ± 1,914.2	5609.2	4.7
SR_medium_	410.3 ± 211.9	439.9	7.2	4221.3 ± 1920.3	3896.7	7.7
SR_slow_	124.9 ± 4.6	137.2	9.8	1009 ± 31.3	1010.0	0.1
Mean ± SD			7.2 ± 2.4			3.5 ± 3.4

## Supplementary Materials

The following are available online at https://www.mdpi.com/1999-4923/11/6/251/s1, Figure S1: Comparison between the observed and the predicted percentage of drug release profiles of sildenafil IR and SR tablets, Figure S2: Comparison between the observed and fitted plasma concentrations of sildenafil obtained after oral administration of the IR and SR tablets in Beagle dogs.
