# Supplementary material for: Physiologically Relevant In Vitro-In Vivo Correlation (IVIVC) Approach for Sildenafil with Site-Dependent Dissolution
Source: Pharmaceutics. 2019 Jun 1;11(6):251. doi: 10.3390/pharmaceutics11060251 (PMC6631943; doi:10.3390/pharmaceutics11060251)
Supplement: Supplementary file 1 [file pharmaceutics-11-00251-s001.pdf]

pharmaceutics

Supplementary Materials: Physiologically Relevant In Vitro-In Vivo Correlation (IVIVC) Approach for Sildenafil with Site-Dependent Dissolution

Tae Hwan Kim, Soyoung Shin, Seok Won Jeong, Jong Bong Lee and Beom Soo Shin

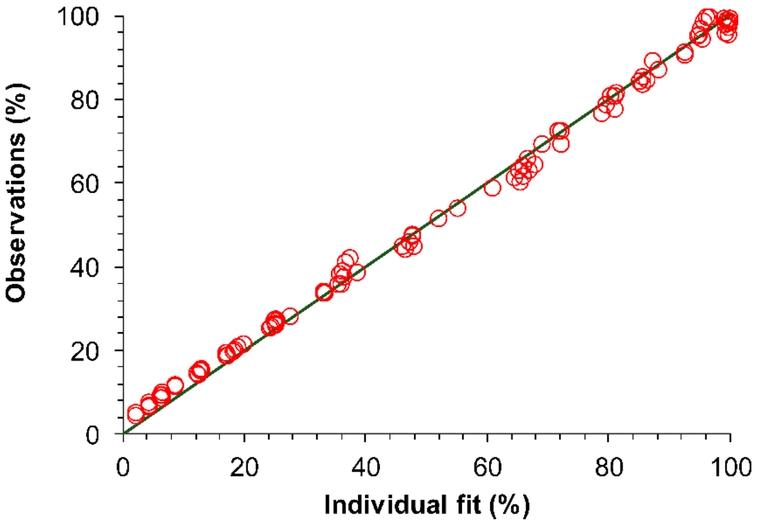

Figure S1. Comparison between the observed and the predicted percentage of drug release profiles of sildenafil IR and SR tablets.

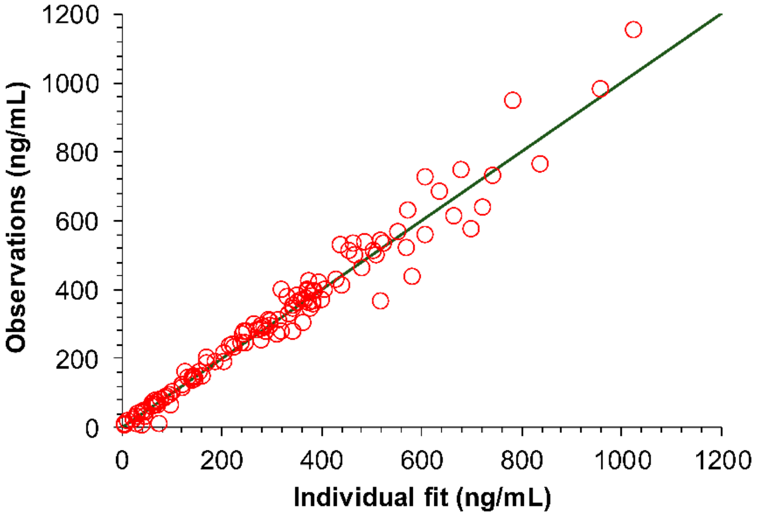

Figure S2. Comparison between the observed and fitted plasma concentrations of sildenafil obtained after oral administration of the IR and SR tablets in Beagle dogs.
